# Collectanea Medica

**Published:** 1814-07

**Authors:** 


### COLLECTANEA MEDICA,

CONSISTING OF

### ANECDOTES, FACTS, EXTRACTS, ILLUSTRATIONS, QUERIES, SUGGESTIONS, &c.

RELATING TO THE

### *History or the Art of Medicine, and the Auxiliary Sciences.*

*Experiment on Respiration, which had nearly proved fatal.—Beneficial Effects of Oxygen Gas in restoring suspended Animation; by* SAMUEL WITTER, Esq. Dublin.

THE following case occurred very lately in the laboratory of the Dublin Society, and excited considerable interest.—When a mixture of carbonate of lime and zinc, or iron filings, is exposed to an intense heat, the peculiar gaseous substance named carbonic oxide is disengaged, which has been stated to bear the same relation to carbonic acid that nitrous gas does to nitric acid. But agreeably to the striking observations of Mr. Higgins, professor of chemistry to the Dublin Society, in his work recently published, wherein his claim to the discovery of the atomic system is unequivocally established, it would appear that, in the combination of oxygen with different gases, it is the atom of oxygen only that is found multiplied, as is beautifully exemplified in all the metallic oxides, acids, and gases. An apparent anomaly has been noticed with respect to nitrous oxide, which the experiments of Mr. Higgins on the composition of nitrous gas tend to obviate, and sanction a comparison of the proportions of carbon and oxygen in carbonic oxide with those of azote and oxygen in nitrous oxide, rather than the atomic coincidence of carbonic oxide and nitrous gas. Carbonic oxide was discovered and described by Mr. Cruickshank in 1801; it is highly combustible, burning with a fine blue flame, but is utterly incapable of supporting animal life.

The diversified experiments of Sir H. Davy on the respiration of nitrous oxide and some other gases, so interestingly described in his scientific researches in 1800, in a great measure dissipated the general apprehensions of fatality resulting from the inhalation of compound gases, and satisfactorily demonstrated that many of the aërial fluids, before considered as destructive to vitality, might be breathed with perfect safety.

Desirous of witnessing the progressive effects of carbonic oxide when freely respired, with a view to comparative analogy in reference to nitrous oxide, I was tempted a few days ago to inhale a portion of it as copiously as possible. The consequence had very nearly proved fatal to me. A considerable quantity of the gas having been carefully prepared by Mr. S. Wharmby, the very ingenious and able assistant to Mr. Professor Higgins, a series of experiments on its respiration were proposed. Mr. Wharmby first noticed some points of resemblance it bore to the nitrous oxide, particularly the singularly sweetish ish taste, and, having made two or three inspirations, was seized with a degree of convulsive tremor and giddiness that nearly overpowered sensibility. These violent effects were but transient, though considerable languor, headache, and debility, remained for many hours afterwards. Anxious to pursue the experiment still further, I next made three or four hearty inspirations of the gas, having first exhausted my lungs of common air as completely as possible. The effects were an inconceivably sudden deprivation of sense and volition. I fell supine and motionless on the floor, and continued in a state of total insensibility for nearly half an hour, apparently lifeless, pulsation being nearly extinct. Several medical gentlemen being present, various means were employed for my restoration, without success; when the introduction of oxygen gas by compression into the lungs was suggested, the effects of which may be fairly contrasted with those of the carbonic oxide. A very rapid return of animation ensued, though accompanied by convulsive agitations, excessive headache, and quick irregular pulsation, and, for some time after mental recovery, total blindness, extreme sickness, and vertigo, with alternations of heat and shivering cold, were painfully experienced. These unfavourable spasms were succeeded by an unconquerable propensity to sleep, which, as might be expected, was broken and feverish. An emetic of tartarised antimony finally removed these alarming symptoms, and the only unpleasant effects felt on the ensuing day were those occasioned by the fall.

I very much regret that the confusion arising from the idea of my death, so disturbed the arrangement that no accurate determination could afterwards be made, either of the quantity of gas respired, or the change it underwent in the process; and the experiment is rather too hazardous for repetition. Nevertheless, the extraordinary efficacy of oxygen gas in cases of suspended animation produced by carbonic acid, choke damps, and other suffocating gases, is fairly deducible, and, I conceive, cannot be too forcibly recommended to the faculty, in such instances. I therefore sincerely hope that the results of this experiment may be of practical utility in those cases, which are so frequently occurring, and are often so awfully fatal; it being the decided opinion of the professional gentlemen present on this occasion, that the free use of the oxygen gas was solely instrumental in restoring me to life.

Mr. Higgins himself had nearly once fallen a victim to a similar experiment with sulphureted hydrogen, the effects of which, after recovering from a death-like insensibility, were painful and oppressive for many days.—*Philosophical Journal.*

---

*The History and Dissection of a Case, in which a Foreign Body was found within the Heart; by* WILLIAM WOOD, Esq. Fellow of the Royal College of Surgeons, Edinburgh.—A. S., a girl of 15 years of age, who had been always of rather a delicate habit, about three years ago, when walking along the street, became suddenly affected with great difficulty of breathing, and general weakness, and was so unwell, that it was with difficulty she could continue her walk walk home. Next day she was rather better, but ever afterwards remained subject to difficulty of breathing on taking any violent exercise. In addition to this complaint, she, soon after the first attack, became affected with constant strong pulsation in the region of the heart, much increased on walking up stairs, or climbing an ascent. It was at this time that I was first called to see her. I found her complaining very much of uneasiness in the chest, from the constant palpitation; she could not bear her clothes at all tight, and she seemed very uncomfortable when the hand was pressed at all firmly on the thorax. The palpitation was so strong, that it could be distinctly seen at a considerable distance. On applying the hand over the region of the heart, strong pulsation was felt; and at a particular spot, about the size of a crown piece, a peculiar thrilling sensation was communicated to the fingers at each stroke of the heart, not easily to be described, but somewhat resembling the effect produced on the ear by the emission of a short hissing sound at frequent regular intervals. She was thin and pale, but took her food well. The pulse was regular. She could lie down in bed, but her sleep was disturbed by disagreeable dreams. These complaints gradually increased in severity, with the addition of fainting-fits of some duration, occurring generally three or four times in the course of every day. These were sometimes so sudden in their attack, that she dropped down instantly, deprived of sense and motion; at other times, according to her friends' account, they were preceded by an involuntary contraction of the fingers and toes. These fainting-fits continued for upwards of two months, when they left her, and never again returned. Being removed to the country, I had no opportunity of seeing her for some time, but I was informed, that, while there, having spit up a considerable quantity of a clear watery fluid, after a severe fit of coughing, she had become considerably easier, and could take a little gentle exercise, which she had not been able to do when she left town. On her return, I found her certainly rather easier, but the strong palpitation was still constantly present. This state of comparative comfort, however, was of short duration, for very soon the symptoms became all much aggravated, with the addition of frequent spitting of blood, though in no great quantity. She was now unable to lie down, but was supported in bed by means of pillows, in nearly an upright posture. The pulse was intermittent. She had a very sallow complexion, and was extremely out of spirits. She lingered on, gradually and slowly losing strength and flesh, till the beginning of November, when she died quite exhausted, above three years from the commencement of her complaints. For many weeks previous to her death, dropsical effusion had taken place into the cellular substance of the lower extremities, and by her friends' account, for about fifteen or sixteen days, large purple spots, of the size of half-crown pieces, had appeared on different parts of her body.

During her illness, digitalis was repeatedly administered, occasionally with the effect of procuring temporary relief; the rest of the treatment treatment consisted in regulating the diet, attending to the state of the bowels, and in occasionally exhibiting cordials.

*Dissection.*—Within the general cavity of the thorax there was found a very considerable quantity of bloody serum. The lungs in general were of a healthy appearance, but towards the root of one of the left lobes, there was discovered a considerable mass of a white gritty concretion.

The pericardium, which was somewhat thickened, being laid open, several ounces of clear serum were removed, and the heart brought into view, considerably larger in size than natural, the increase appearing most remarkable on the right side, the cavities of which, particularly the auricle, were much distended with blood. The heart being removed from the body, with the view of having it particularly examined, the right auricle and ventricle were laid open; in these the disease seemed to be entirely confined to enlargement of their cavities. The tricuspid and semilunar valves of the pulmonary artery were apparently in a sound state.

On slitting open the left auricle, which appeared larger than usual, the musculi pectinati were observed to be considerably increased in thickness; and there was found occupying, and nearly entirely filling up the cavity of the left sinus venosus, a round firm body of considerable size; immediately under which, and over the situation of the opening into the ventricle, there was contained another solid substance of another form. A third substance, rough and irregular, had also been lodged in the auricle, but its situation could not be precisely ascertained; it lay however towards the ventricular opening. All these being removed, an attempt was made to pass the finger from the auricle into the ventricle, but this was found to be impossible. The left ventricle was therefore fully laid open by an incision, and, on examining the mitral valves, they were found to be much thickened, and quite opake, by their connection and situation permanently contracting the opening from the auricle into a small oval fissure, into which, not even the tip of the little finger could be introduced.

The semilunar valves of the aorta were opake, and much thickened.

The substance, occupying the sinus venosus of the left auricle, when particularly examined, was found to be of a darkish red colour, in form completely spherical, measuring rather more than an inch and a half in diameter. It felt firm, but elastic; the surface was every where smooth and polished but having a singularly clotted appearance. It had no connection with the surrounding parts, rolling loosely in the auricle. When cut open, after having been kept for some days in diluted alcohol, it was found to consist of a sac, one eighth of an inch in thickness, formed of an immense number of firm smooth laminæ, which could be easily separated from each other. Within the cavity formed by this sac, was contained a quantity of coagulated blood. The flat substance which lay between the round body and the opening into the ventricle, was of an oval form, an inch in length, and three-fourths of an inch across at the broadest part. Its upper surface was of the same smooth clotted appearance with that of the former, slightly concave, so as to form a kind of superficial socket for it to roll in; the under surface, which lay over the mitral valves, was rough and irregular, containing something resembling a firm coagulum of blood in a concave depression.

The symptoms and progress of the disease in this case, differ little from those generally met with in cases where there has been found, after death, such an alteration in the structure of the heart, or of the large vessels arising from it, as must have disturbed the regularity of the circulation of the blood through that organ; the dissection, however, appears to be singular and interesting, from its affording an example of the formation, during life, and existence for probably a great length of time, of loose solid substances, of considerable size, within one of the cavities of the heart.

All the symptoms of the disease, as well as the morbid appearances of the right cavities of the heart, may be, I think, satisfactorily explained on the principle of obstruction to the free passage of the blood through the left side of that organ; and, indeed, the principal difficulty in the case seems to be, in conceiving how the circulation should have been sufficiently carried on to maintain life for any length of time, under such a degree of obstruction as must have been produced in this instance, by the joint effect of the diseased mitral valves, and of the foreign bodies contained in the left auricle, the former materially contracting the opening between the auricle and ventricle, and the latter retarding and diminishing the current of blood towards it; an effect which, from their situation, size, and form, these substances seem to have been well calculated to produce.

With regard to the morbid appearances on the left side of the heart, the most probable supposition appears to me to be, that the disease had originally existed in the mitral valves, producing a diminution in the size of the ventricular opening; that, in consequence of this contraction, accumulation of blood had taken place in the left auricle, and coagula had been there formed, upon which coagulable lymph had been afterwards slowly and gradually deposited; the different substances thus formed assuming different shapes, according to the relative situations in which they had been placed with regard to each other, and to the internal parts of the auricle. But whether this be or be not the true account of the formation of these solid bodies, there can be no doubt, I think, from their laminated structure, that they had existed during life; indeed, upon even the most superficial examination, they are found to be essentially different from those coagula so frequently formed after death in the different cavities of the heart, termed polypi. They are quite different too from those apparently organized solid substances, which have been occasionally met with, adhering firmly to some part of the internal surface of the heart.—(Allan Burns on the Diseases of the Heart.)

That cases in which foreign bodies have been formed within the heart heart during life are of rare occurrence, may be inferred from the circumstance of Dr. Baillie not having seen one of the kind. In the fourth edition, however, of his invaluable work on Morbid Anatomy, he refers, in a note, to two cases which had occurred to Mr. Brodie. "In some instances," says Dr. Baillie, "a coagulum of blood has been found of a laminated texture, in such parts of the heart as are most remote from the direct current of the circulation. This laminated texture shews, that the coagulation had taken place during life, and in a gradual manner. Two cases of this kind have been observed by Mr. Brodie, who, although young, is already well known to the public as an excellent anatomist and physiologist. Such cases, however, are to be considered as different from the coagulations which have been generally called polypi, and which fill up entirely one or more of the large cavities of the heart."—(Note to the fourth edition of Baillie's Morbid Anatomy.)

From the concise account here given of these substances, they appear to agree with those met with in the present instance, in being of a laminated texture; they differ, however, with regard to situation, in Mr. Brodie's cases having been placed out of the direct current of the circulation, while here they must have been constantly and directly exposed to the stream of blood from the pulmonary veins. Portal makes mention of various kinds of substances found within the heart, some of which he says, are of a laminated texture. He seems to doubt, however, their having been formed during life, although he does not deny the possibility of such a thing happening.

My friends, Dr. Gordon and Mr. Turner, inform me, that they recollect seeing, at a dissection in the Royal Infirmary here, five small loose round substances, of a rough shaggy appearance, taken out of the left auricle, in a case in which there had existed disease of the mitral valves; when opened they were found to be hollow, and to contain a quantity of a whitish fluid.—*Edinburgh Med. and Surg. Journal.*

---

*Case of a Man who vomited a urinous-tasted Liquid;* by W. Reid Clanny, M.D. M.R.I.A. Honorary Member of the Royal Physical Society of Edinburgh, and Physician to the Sunderland Dispensary.—Ralph Cooper, æt. 24, Ph. Pulmonalis et anasarca. Admitted at the Sunderland Dispensary, May 27th, 1813. This young man is well formed but delicate. His feet and legs are much swelled and hard; the urine is in very small quantity and high coloured; the pulse is frequent and quick in the beat. He is tormented by a severe cough and purulent expectoration; and not long since the sputum was mixed with blood, and frequently the hæmoptysis has been severe. By the use of digitalis purpurea in substance, supertart. potassæ, diluted sulphuric acid, and occasional opiates and laxatives, he has been greatly relieved, though the relief is not permanent; for the least exposure to cold or damp produces a return of all his bad symptoms.

It is not needful to record here the daily and weekly practice; as it would not only greatly enlarge the communication, but also distract the attention from that particular phenomenon to which I wish to draw the readers. I was called to visit him upon the 12th of December, when he informed me that from the medicines which I had ordered him he was so much relieved, that he considered himself as restored to health, and that a few days before he had been working very hard as a caster of coals upon the river Wear, which had produced a return of his former complaints to a much greater degree than hitherto. His legs and thighs are considerably swelled, the abdomen is much distended, and shows all the symptoms of ascites abdominalis. The urine does not exceed a pint in quantity, which is very high coloured, and upon cooling a lateritious sediment is deposited. The bowels are pretty regular, and the pulse fluctuates between 115 and 120. With these symptoms, the expectoration is purulent and copious. The appetite is very bad and frequently depraved. In a word, there is not one favourable symptom, and his dissolution may be expected at no very distant period. It is his wish, as well as mine, to obviate particular symptoms, so as to render him comfortable.

20th December.—Since my last visit he has been attacked several times by severe and incessant vomiting: he observed that the quantity of liquid which he vomited greatly exceeded the quantity of liquid which he had swallowed for several days before; while at the same time the urine which was passed by the urethra was stationary as to quantity, and tolerably healthy in appearance. He describes the liquid which he vomited as exceedingly offensive, and to be of a salt and urinous taste. He was astonished to find that the feet, legs, thighs, and abdomen, gradually diminished in size during the time of the vomiting, which must have been affected solely by the vomiting, in seven days; for, as was stated above, he was then using no active remedies, which appeared to be quite unnecessary in his very deplorable state. He also reports that the quantity of water which came off his stomach must have been to the extent of several gallons. The bowels have been and still continue regular.

Under these circumstances I became an anxious spectator, except that, when needful, antispasmodics were directed to alleviate the very uncomfortable state of the stomach.

January 5, 1814.—This day I found the patient relieved from all dropsical symptoms, but in a state of great debility; pulse 82, weak, soft, and regular. He is able to sit up in the afternoons. The urine is in sufficient quantity and approaches a natural state. He is not thirsty as formerly, though he finds it needful to rinse the mouth at different times in the day with tamarind-water. His appetite is very bad, and he spirts urine to a considerable extent, without any difficulty. He remarks that after the distressing vomiting ceased, he was tormented by a severe itching over the whole body, particularly upon the hands, which now appear as if they were affected by an herpetic eruption. He has no uneasiness of the thorax, but from indigestion he has pain in the region of the stomach The vomiting is at times troublesome, though he does not observe that there is more liquid vomited than he had previously swallowed. A

distressing distressing diarrhœa has commenced, which is occasionally relieved by opium pills.

It is much to be regretted, that I had it not in my power to examine the liquid which he vomited in such large quantities, as a chemical analysis would have demonstrated its affinity to urine; but from not knowing of the very singular and unlooked-for turn which took place, till after the vomiting had ceased, such a desirable circumstance was entirely frustrated.

The only cases of a similar nature which I have read, are to be found in Dr. Percival's Essays, Medical, Philosophical, and Experimental, at page 375, vol. 1st, 4th edition; where the case of a female is narrated in his easy, natural, and unaffected style. In the Medical Facts and Observations, vol. 6, page 212, the case of a female is well delineated; besides these, the case of a nun and of a monk, are to be found in the Histoire de l'Academie Royale des Sciences, Années 1715 and 1722. But in none of these cases is any mention made of the salt and urinous taste of the liquid which was vomited, as in the case of Ralph Cooper; and here the theory of "the regurgitation of fluids in the absorbent vessels," which was broached by that superior youth, Mr. Charles Darwin, naturally presents itself to the mind: of his theory, and the experiment made upon his friend, an ample account is to be found in that excellent practical work the Medical Commentaries, vol. 7, page 193. *Annals of Philosophy.*

---

*Case of Retention of Urine successfully treated by puncturing the Bladder;* by JOHN TAUNTON, Esq. Surgeon to the City and Finsbury Dispensaries, and to the City of London Truss Society for the Relief of the ruptured Poor, &c. &c.—John Jones, aged 67, a brass turner by trade, a stout muscular man, his general health extremely good, with the exception of an ulcerated leg for the last twenty years, and, during the last eight or ten, hydrocele on one side and hernia on the other. For the last twelve months also he has experienced some difficulty in voiding his urine, which came away in small quantities at a time, and with frequent calls. On July 7, 1813, after a hearty dinner he sat an hour or two in the open air, during which time he was attacked by violent pain in the abdomen, with purging. These symptoms continued all night, and his urine came away in drops without any effort. On the 8th he was admitted a patient of the Finsbury Dispensary. The abdomen was hard, swelled, and painful; and there was a considerable degree of fever, attended with thirst; the purging also continued. The leg, which had been much inflamed for the last three months, is now better. An anodyne fomentation and some powdered rhubarb were prescribed for him by the physician who visited him till the 13th; and during the interval aperient fomentations, &c. were resorted to with a view to relieve the complaint in the bowels and general disorder of the constitution. On the 13th, Mr. Taunton was requested to see him, on account of the stillicidium urinæ which had existed

 from from the beginning of the attack. He found him with quick weak pulse, brown tongue, violent pain in the region of the bladder, which was distended, forming a tumour reaching above the umbilicus; the urine was dribbling away involuntarily. The catheter was introduced, but could not be passed beyond the neck of the bladder. The smallest gum catheters were also tried without effect. Continuetur fomentum.

14th.—The bladder reaches still higher up; the catheter again attempted, but in vain; other symptoms the same. Puncturing above the pubis was determined on, and consented to by the patient. Upon going to perform the operation at three o'clock, it was found that a considerable quantity of urine had come away involuntarily, and almost in a stream, and the patient would not now consent to the operation: nor was it urged, as the bladder was greatly reduced in size. The tongue was still brown, and the other bad symptoms continued.

15th.—The patient was nearly in the same state: stillicidium constant.

16th.—The tumour formed by the bladder is more prominent and circumscribed; reaches about two inches above the umbilicus; in other respects the same: pain decreased. The operation was now performed, and between two and three quarts of urine were taken away; it was not grumous, nor materially altered from that of a healthy person. During the operation the pulse fell, but soon regained its strength. A long elastic catheter was left in the wound, and properly secured. A cordial mixture was prescribed. In the evening the bougie used as a stilette was withdrawn, and the urine evacuated: no pain on pressure on the abdomen, which was soft, and the tongue clean.

18th.—The urine escapes by the side of the catheter, but is not effused into the cellular membrane; adhesive inflammation was visible round the wound. The urine flows involuntarily, but he feels easy. He took broth yesterday: the tongue was clean, the pulse was stronger and slower.

19th and 20th.—A slight blush of inflammation immediately around the wound; no pain experienced on pressure.

21st.—Has felt pain in the night, seemingly from a temporary obstruction to the flow of urine, which was soon evacuated, and the pain went off.

22d.—The catheter escaped during the night; but the urine flows freely from the orifice, and he continues to gain strength. From this period to August 28th convalescent. The passage of a small bougie has been attempted two or three times without success. The patient complains of considerable pain in the urethra, which prevents his sleeping without opiates; and he takes a grain of opium every night. He walks out, and his spirits are better.

September 10th.—The quantity of urine discharged by the urethra has sensibly increased, until it all comes away by that channel. The opening had closed a few days before, but it broke out when straining straining at stool; he does not know whether any urine escaped or not, but nothing comes from it at present.

17th.—Much in the same state; complains of soreness where the puncture was made, and a little matter oozes from it; a considerable quantity of urine came through on the 13th and 14th. From this time he gradually recovered, and was discharged cured the beginning of November, since which he has not had any return of the complaint.—*Philosophical Journal.*

---

*Copy of a Letter from* JOSEPH D. A. GILPIN, M.D. *Inspector of Military Hospitals at Gibraltar, to* COLIN CHISHOLM, M.D. F.R.S. *&c. Bristol.*

My dear Sir,—Your letter of April last must have taken a long round before it reached me.

I have lately seen Dr. Bancroft's book on Yellow Fever; and was sorry to observe, that a difference of opinion could be productive of a harshness of language and remark, surely inadmissible, on a topic which merely demands candid investigation, and fair argument

Of the infected state of the Hankey, I never did, nor ever shall entertain the least doubt; nor do I recollect that any medical man in Grenada held an opposite opinion. During a residence of eighteen years in that island, I had frequent opportunities of seeing the yellow remitting fever; but I have not the least remembrance, that, at any period previous to the arrival of the ship in question, a suspicion even was entertained of its being contagious. During a lapse too of so many years, the state of the atmosphere, and the prevalence of marsh miasmata, must have frequently been as they were in 1793, and should consequently (the causes being the same) have produced similar effects.

Soon after the arrival of the Hankey, a fever appeared, attended by the symptoms so accurately detailed in the 6th chapter of your first volume. Those which always struck me as being peculiar to the disease, were, the appearance of the eye altogether, with the dilated pupil, and exquisite pain felt at the bottom of its orbit. The rapid fatal termination, too, often in 36 hours after its attack, I do not remember to have witnessed in the bilious remittent of tropical climates.

I was not present at the examination of Captain Cox by the Lieutenant-Governor; but I perfectly recollect its result being mentioned to me: namely, that he had kept the clothes and bedding of his passengers.

That those who had visited the Hankey brought the contagion into the town of St. George, and that it spread from thence into the country, I have as little doubt as I have of my present existence; and I really believe, that had the most determined anti-contagionist been present during that awful period, he would have become a proselyte to the general opinion.

At Martinique, in 1794, we suffered dreadfully from a fever in every respect similar to that which I had seen in Grenada, the preceding year. And in numberless instances, its contagious nature was manifested, by its attacking those who were, *immediately*, about the sick.

sick. We had, in the General Hospital at St. Pierre's, some hundred cases of it; and it is a melancholy truth, that very few of the attending medical officers survived the pestilent duty in which they were employed.

A most striking instance of contagion, among many others, occurred after the short sickness of the wife of a staff-surgeon. She arrived at Martinique from Barbadoes, and remained on-board the vessel a day or two before a lodging could be procured for her. In the mean time she dined with us once at the General Hospital, and returned in the evening to sleep in the ship. The day after, she was seized with the fever in question, and it terminated fatally within 48 hours of its attack. Her immediate attendants were four servants, and I was frequently with her during the day and night. The servants were speedily affected, and died within as short a period as their mistress; and I was the only one who survived of those who were about her person. The captain of the vessel (in which she came) with those who assisted in getting her out of the ship, and in conveying her to her lodgings, died a few days after her decease; and I was credibly informed, that, shortly after, the crew died to a man. I lay no stress, however, on this hear-say evidence; what I witnessed myself carried an indelible conviction to my mind of the highly contagious nature of the disease.

With regard to the supposed origin of the fever that raged in this garrison, and which carried off nearly five thousand persons, in the year 1804, I cannot say any thing of my own knowledge, but I have now before me a description of it, drawn up by a very acute and accurate observer, who was on the spot. He was present during the examination of a young man named Santos, who declared as follows: "That he arrived from Cadiz at Gibraltar, on the 25th of August, 1804; and that two days afterwards, he was seized with a fever, and that while he was ill, his mother, two of his aunts, a brother, and two sisters, who were in the same house with him, were taken ill also of fever, of which his mother and his aunts died on the 16th of September. He confessed, that, when in Cadiz, he was in a room with a person labouring under a fever." A person of the name of Pratts arrived also in the garrison, who had been ill of fever while living in the same house at Cadiz with Santos. He was the master cooper of the naval victualling yard, and of fair character. His examination was taken on oath, and what follows is an extract from it: "That he arrived at Cadiz on the 3d or 4th of August, 1804, and lodged in the tavern Del Sols, where he remained fifteen days. He was taken ill of fever, and continued so upwards of a week. That he had black vomiting. On his recovery his yellow appearance induced a captain of a vessel to refuse him a passage. That the captain of his own privateer, with himself, lived for many days in the same tavern with a man named Santos; and that he was in the same room with him when he (Pratts) was ill. That while he was in the tavern, several persons were ill. That he was attended after his removal from the tavern by a man to whom he gave some of his clothes; and that shortly after wearing them, he fell ill and died." The examination of Santos, and the above above deposition, with the death of those in the house with Santos, convince me that the fever originated in this garrison from him; and from what I have heard since my arrival here, and from what I have seen of bilious remittent fevers, during two summers in the hospitals, I firmly believe, though in opposition to the opinions of very highly respectable medical characters, that the disease in this garrison in 1804 was contagious; and that its symptoms were similar to those which attended the fever of 1793, in the island of Grenada.

I know not what might formerly have been the state of the lower lands beneath this rock; but at present, as far as my observation goes, few places are less liable, I should think, to suffer from marsh miasmata, than Gibraltar.—*Edinburgh Med. and Surg. Journal.*

